# Potential recombination between SARS-CoV-2 and MERS-CoV: calls for the development of Pan-CoV vaccines

**DOI:** 10.1038/s41392-023-01396-6

**Published:** 2023-03-15

**Authors:** Qiao Wang, Lujia Sun, Shibo Jiang

**Affiliations:** Key Laboratory of Medical Molecular Virology (MOE/NHC/CAMS), Shanghai Institute of Infectious Disease and Biosecurity, School of Basic Medical Sciences, Fudan University, 131 Dong An Rd, Xuhui District, Shanghai, 200032 China

**Keywords:** Vaccines, Infectious diseases

In a recent study published in *Nature*, Yan and colleagues demonstrated that NeoCoV, a potential MERS-CoV ancestor in bats, can use bat ACE2 as its entry receptor and that NeoCoV S pseudotyped virus, which contains a T510F mutation in the receptor-binding domain (RBD), enters cells expressing human ACE2 (hACE2).^1^ The findings reveal a potential zoonotic threat that MERS-CoV could, during its evolution, be gaining the ability to use hACE2 as an entry receptor and, thus, could also coinfect ACE2-expressing cells with SARS-CoV-2.

**Fig. 1:**
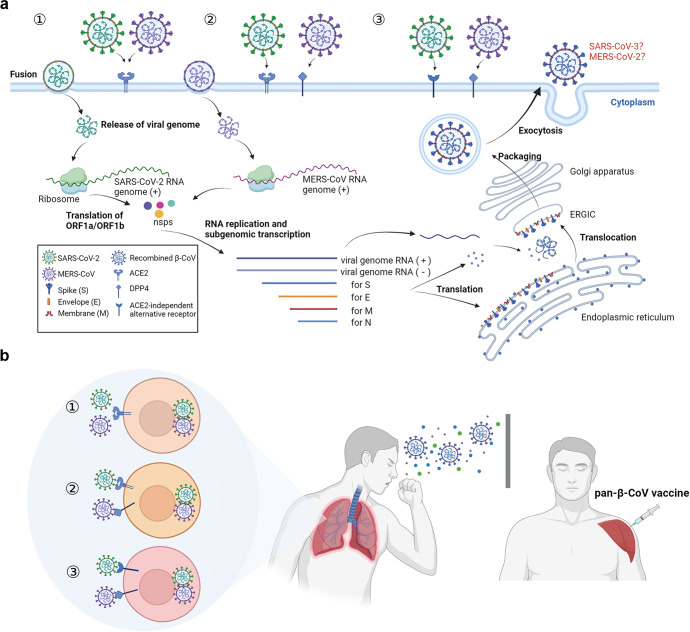
Coinfection of SARS-CoV-2 and MERS-CoV may result in the emergence of recombined β-CoV, SARS-CoV-3, or MERS-CoV-2, thus calling for the development of pan-CoV vaccine. **a** Because of the identical infection cycle of SARS-CoV-2 and MERS-CoV, recombination may occur when ACE2-using MERS-CoV coinfect with SARS-CoV-2 in ACE2-expressing cells ①, or when SARS-CoV-2 and MERS-CoV coinfect a cell expressing both ACE2 and DPP4 ②, or when MERS-CoV coinfect with SARS-CoV-2 in the cells expressing DPP4 and one of the ACE2-independent alternative receptors (such as CD147, NRP1, ASGR1, KREMEN1, and AXL)③, resulting in an emerging β-CoV, SARS-CoV-3 or MERS-CoV-2, via genetic recombination between SARS-CoV-2 and MERS-CoV. (**b)** SARS-CoV-2/MERS-CoV recombination calls for the development of a pan-β-CoV vaccine to prevent infection of the newly emerged β-CoV, SARS-CoV-3, or MERS-CoV-2. The illustration was created by the author (Lujia Sun) using BioRender program (http://www.biorender.com)

It is generally believed that sarbecoviruses, such as SARS-CoV-2 and SARS-CoV, use angiotensin-converting enzyme 2 (ACE2) as the entry receptor, while merbecoviruses, such as MERS-CoV, HKU4, and HKU25, utilize dipeptidyl peptidase 4 (DPP4) as the entry receptor. Some alveolar cells and small intestinal cells in the human body can express both ACE2 and DPP4, providing an opportunity for coinfection by both SARS-CoV-2 and MERS-CoV. With an even greater span of infectivity, SARS-CoV-2 may use its alternative receptors, such as CD147, NRP1, ASGR1, KREMEN1, or AXL, to enter host cells and may coinfect with MERS-CoV in cells expressing DPP4 and one of the alternative receptors (Fig. 1a). Moreover, these β-CoVs may use identical transcriptional regulatory and conserved sequences upstream of the open reading frame to mediate discontinuous transcription of the viral genome, possibly resulting in recombination of SARS-CoV-2 and MERS-CoV.

Indeed, genetic recombination between different coronaviruses has been well documented. For example, the MERS-CoV outbreak in 2015 resulted from genetic recombination among diverse MERS-CoV lineages.^2^ Also, SARS-CoV-2 recombinant lineages XD and XE were generated through the recombination of SARS-CoV-2 Delta and Omicron variants and Omicron subvariants BA.1 and BA.2, respectively. In addition, Omicron subvariant XBB emerged via recombination of Omicron BA.2 subvariants BJ.1 and BM.1.1. Therefore, recombination among coronaviruses is a well-established phenomenon.

Even as SARS-CoV-2 continues its worldwide spread, MERS-CoV remains a threat. MERS-CoV is the most virulent human pathogenic coronavirus known to date, even though only sporadic infections have been reported in the Middle East since 2016. Most recently, it was warned that the Qatar FIFA World Cup 2022 and camel pageant championships might increase the risk of MERS-CoV transmission and global spread.^3^ In 2015, a similar instance occurred with an individual returning to Seoul from Saudi, resulting in 184 infections with 36 deaths.^2^ As a reminder, no MERS vaccine is available, even while the case-fatality rate for MERS-CoV infection is astonishingly high, approximately 35%, 100 times higher than that for COVID-19.

Even more concerning is the risk of a newly emerging β-CoV clade, SARS-CoV-3 or MERS-CoV-2, via genetic recombination between SARS-CoV-2, particularly the Omicron subvariant, and MERS-CoV. Furthermore, it is likely that such a new β-CoV clade may bear high SARS-CoV-2-like transmissibility along with a high MERS-CoV-like case-fatality rate, which would have catastrophic repercussions.

Thus, receptor-sharing by SARS-CoV-2 and MERS-CoV relatives makes simultaneous infection by two different coronavirus clades more likely, even leading to a higher probability of RNA-RNA recombination within the same cell. Several cases of SARS-CoV-2/MERS-CoV coinfection were recently reported in Saudi Arabia,^4^ possibly foreshadowing recombination between these two β-coronaviruses. Thus, in places still suffering from MERS-CoV infection, it is particularly necessary to use real-time quantitative PCR for MERS-CoV screening in patients with SARS-CoV-2 infection.

The mounting likelihood of SARS-CoV-2/MERS-CoV recombination calls for the development of broad-spectrum vaccines and therapeutic drugs with efficacy against pan-β-coronaviruses, especially those sharing the same host receptor (Fig. 1b). Many research teams have attempted to develop pan-β-CoV vaccines. For example, researchers at DIOSynVax used 3D computer modeling to design vaccine antigen payloads and deployed them in mRNA-, virus vector-, or protein-based β-CoV vaccines.^5^

Vaccines containing multiple conserved neutralizing antibody epitopes or T cell epitopes from different β-CoVs, but simultaneously displayed on one kind of carrier, such as nanoparticles, are expected to elicit cross-reactive immune responses against multiple β-CoVs. Our team has identified a STING antagonist-based adjuvant, termed CF501,^5^ that can be used for the development of highly effective and long-lasting β-CoV vaccines.

The two broad-spectrum neutralizing antibodies B6 and S2P6 targeting a conserved stem helix epitope in the S2 subunit of SARS-CoV-2 spike protein are also effective against Neo-CoV infection, suggesting that antigens containing the stem helix region may be used for the development of pan-β-CoV vaccines.^1^ Another broad-spectrum neutralizing antibody, 76E1, was found to target a highly conserved S2’ cleavage site and fusion peptide epitope within the S2 subunit, indicating that these regions can also be used for the development of pan-β-CoV vaccines.^5^

In sum, given the high risk of SARS-CoV-2/MERS-CoV recombination, the development of pan-β-CoV vaccines as well as CoV entry and replication inhibitor-based therapeutics is urgently needed to combat the pandemics or epidemics caused by emerging SARS-CoV-3 or MERS-CoV-2 in the future.
